# Allele-specific suppression of pathogenic bestrophin-1 transcripts by CRISPR/Cas9-mediated genome editing

**DOI:** 10.1186/s13073-026-01649-3

**Published:** 2026-04-20

**Authors:** Andrea Milenkovic, Bernhard H. F. Weber

**Affiliations:** 1 Institute of Human Genetics, University of Regensburg, Universitätsstrasse 31, Regensburg, D-93053 Germany; 2 Institute of Clinical Human Genetics, University Hospital Regensburg, Franz-Josef-Strauß-Allee 11, Regensburg, 93053 Germany

**Keywords:** Bestrophin-1, BEST1, Allele-specific targeting, CRISPR/Cas9, Dominant-negative mutation, BVMD, Best disease, Autosomal dominant retinal disorder

## Abstract

**Background:**

Treating autosomal dominant gene mutations remains challenging, particularly when mutations convey a gain-of-function or a dominant-negative effect, as standard gene supplementation strategies often fail to counteract the pathogenic allele.

**Methods:**

In this study, we employed human induced pluripotent stem cell-derived retinal pigment epithelium (hiPSC-RPE) to investigate allele-specific CRISPR/Cas9 genome editing as a potential treatment for Best disease (BD), an autosomal dominant macular dystrophy caused by over 250 distinct mutations in the bestrophin-1 (BEST1) gene. We designed and evaluated single guide RNAs (sgRNA) targeting three known BEST1 mutations (p.(R218C), p.(A243V), and p.(I295del)), assessing their impact on BD-associated hiPSC-RPE phenotypes and BEST1 channel function. Computationally predicted sgRNAs were rigorously tested for on-target efficiency, allele specificity and genome-wide off-target activities.

**Results:**

We found that shortening sgRNA length improved specificity in some cases, while introducing an additional mismatch generally compromised editing efficiency. Notably, only one of the three mutations yielded an sgRNA with both high cleavage efficiency and undetectable off-target effects in hiPSC-RPE cells. We then explored the consequences of allele-specific editing on *BEST1* expression and function in clonal BD hiPSC-RPE lines. Eliminating the mutant *BEST1* transcript led to enhanced BEST1 localization, improved protein stability and restoration of anion transport function.

**Conclusions:**

Taken together, our findings support allele-specific gene editing as a viable therapeutic strategy for selected BEST1 mutations, while underscoring the necessity for rigorous testing of computationally designed sgRNAs, given their mutation- and context-dependent variability.

**Supplementary Information:**

The online version contains supplementary material available at 10.1186/s13073-026-01649-3.

## Background

Bestrophin-1 (*BEST1*) is one of four paralogous genes in the human bestrophin family [1]. It encodes an integral membrane protein, that is predominantly localized to the basolateral membrane of the human retinal pigment epithelium (RPE) [2]. Pathogenic variants in *BEST1* have been implicated in at least three clinically distinct retinopathies, collectively referred to as bestrophinopathies. These include the autosomal dominant forms Best vitelliform macular dystrophy (BVMD), also known as Best disease (BD) (MIM 153700) [3, 4] and autosomal dominant vitreoretinochoroidopathy (MIM 193220) [5], as well as the autosomal recessive bestrophinopathy (MIM 611809) [6].

Among these, BD is the most prevalent and is characterized by early onset accumulation of lipofuscin-like deposits in the macula, leading to progressive central vision loss. A distinguishing electrophysiological feature of bestrophinopathies is the markedly abnormal electrooculogram, specifically a reduced Arden ratio (light peak/dark trough ratio), likely reflecting impaired BEST1-mediated Ca^2+^-activated chloride channel activity [7–10].

Dominant-negative mutations in *BEST1* have been identified as a primary cause of BD [11–15]. These mutations disrupt BEST1 function through multiple mechanisms, including mislocalization of the protein within the RPE [11, 12, 16], altered ion gating properties [7, 17–19], and reduced protein stability [13, 20]. Collectively, these effects lead to impaired chloride ion transport, a hallmark of BEST1-associated dysfunction. To date, over 250 distinct pathogenic mutations in *BEST1* have been reported.

Structural studies using X-ray crystallography and cryo-electron microscopy of chicken and human BEST1-Fab complexes have revealed that the eukaryotic BEST1 channel adopts a pentameric architecture, composed of five homomeric BEST1-subunits [21–23]. These subunits are thought to mediate multiple physiological roles, functioning not only as Ca²⁺-activated chloride channels but also as GABA receptors and neurotransmitter-conducting channels, the latter role recently reported by Wang et al. [24].

The dominant-negative effect of most pathogenic mutations is likely due to the oligomeric channel structure. Incorporation of even a single defective subunit can compromise the function of the entire pentamer. Consequently, allele-specific silencing that selectively ablates the mutant allele while preserving the wildtype copy may represent an attractive therapeutic strategy. Such considerations have already demonstrated efficacy at both the RNA and protein levels in several genes [25, 26].

Recent advances in CRISPR/Cas technology have enabled the allele-specific targeting of disease-causing single-nucleotide variants in various cell models, including patient-derived induced pluripotent stem cells (hiPSCs) [14, 15, 27–29], patient fibroblasts [30, 31], and cancer cell lines [32, 33]. By incorporating the mutant base within the 17–20 nucleotide (nt) protospacer region of the single guide RNA (sgRNA), Cas9 nuclease activity can be selectively directed to the disease allele, inducing double-strand breaks (DSBs) and disrupting expression of the pathogenic variant. Importantly, this strategy has also been translated into animal models of human disease, demonstrating for example phenotypic rescue in the P23H rhodopsin knock-in mouse, a model for autosomal dominant retinitis pigmentosa [34], the Beethoven mouse model for autosomal dominant deafness [35], and a mouse model of Duchenne muscular dystrophy [32, 36].

Currently, no approved treatment exists for BD patients. To support the clinical translation of a mutation-specific CRISPR/Cas9 approach for BD, our study rigorously evaluated computationally designed sgRNAs targeting three pathogenic *BEST1* mutations. We further sought to validate the targeted knockout of the mutant *BEST1* allele and assess its impact on RPE cell phenotype and BEST1 channel function. We demonstrate that reduced *BEST1* gene dosage with only one functional allele remaining is sufficient to maintain physiological *BEST1* activity in the RPE, the primary site of BD pathology.

## Methods

### Antibodies

For immunocytochemistry (ICC) and Western blotting (WB), the in-house rabbit polyclonal primary antibody hBEST1-334 was used at dilutions of 1:250 ICC and 1:2,500 WB, as previously described by Milenkovic et al. [11]. Commercial monoclonal mouse antibodies included β-catenin (ICC, 1:1000, #610153, BD Biosciences, Franklin Lakes, USA) and β-actin (WB, 1:10,000, #A5441, Sigma-Aldrich, St. Louis, USA). Secondary antibodies for WB were goat anti-rabbit or anti-mouse IgGs conjugated to near-infrared IRDye fluorophores (1:10,000, LI-COR Biosciences, Bad Homburg, Germany). For immunofluorescence, secondary antibodies included Alexa Fluor 594-conjugated goat anti-rabbit and Alexa Fluor 488-conjugated goat anti-mouse (both 1:500, Thermo Fisher Scientific, Waltham, USA).

### DNA Extraction, PCR amplification, and Sanger sequencing

Genomic DNA (gDNA) was extracted from hiPSCs and hiPSC-RPE cells using the Nucleospin^®^ Tissue Kit (MACHEREY-NAGEL GmbH & Co. KG, Düren, Germany). PCR amplification of both gDNA and complementary DNA (cDNA) was performed using GoTaq^®^ DNA Polymerase (#M317, Promega, Mannheim, Germany). Sanger sequencing was carried out using the BigDye Terminator v1.1 and v3.1 Cycle Sequencing Kits (Life Technologies, Carlsbad, USA). Sequencing reactions were analyzed on an ABI 3130 × 1 Genetic Analyzer (Applied Biosystems, Thermo Scientific, Waltham, USA). Oligonucleotide primer sequences are listed in Additional File 1: Table S1.

### Cohort details, hiPSC generation, and RPE cell differentiation

In the current study, two sets of iPSC lines were used. The first set comprised hiPSC lines drived from two unrelated healthy donors (MK_control_ and AM_control_) and four patient- derived iPSC lines (MO_⁺/R218C_, SK_⁺/A243V_, MD_⁺/I295del_ and AP_⁺/I295del_) heterozygous for *BEST1* mutations p.(A243V), p.(I295del), and p.(R218C). The lines have been described in detail previously [13]. The second set consisted of six iPSC lines generated following electroporation of the sgRNA/Cas9 complex into the parental MD_⁺/I295del_ and AP_⁺/I295del_ lines. This set included four CRISPR/Cas9-edited clones (AP_crispr#16_, AP_crispr#19_, MD_crispr#2_ and MD_crispr#22_) carrying DSB-induced frameshift mutations as well as two iPSC clones (MD_+/I295del__#1 and MD_+/I295del__#3) derived from Cas9-electroporated MD_⁺/I295del_ cells that showed no detectable genome editing. Detailed information of the iPSC lines used is specified in Additional File 1: Table S2. An overview of hiPSC clones used for each experiment is given in Additional File 1: Table S3. All hiPSCs lines were derived from fibroblasts.

The generation and differentiation protocol was adapted with minor modifications from Brandl et al. [37]. Briefly, all hiPSCs were reprogrammed from human fibroblasts via electroporation with a combination of episomal plasmids pCBX-EBNA, pCE-hSK, pCE-hUL, pCE-hOCT3/4, and pCE-mp53DD, as described by Okita et al. [38]. All plasmids were originally provided by Shinya Yamanaka and obtained through Addgene (plasmids #41857, #41814, #41855, #41813, and #41856, Addgene, Massachusetts, USA). Electroporation of 500,000 human fibroblasts was performed using the Amaxa Nucleofector system (program B-016) in combination with the Human Dermal Fibroblast Nucleofector™ Kit VPD-1001 (Lonza, Basel, Switzerland), following the manufacturer’s instructions. Pluripotency of the resulting hiPSC lines was confirmed by RT-PCR analysis of pluripotency-associated marker mRNA expression (see Additional file 2: Fig. S1). Oligonucleotide primer sequences are listed in Additional File 1: Table S4.

For differentiation into RPE cells, hiPSC-derived progenitors were seeded onto six-well Corning Transwell^®^ filter inserts (0.4 μm pore size, 657641, Greiner, Kremsmünster, Austria), pre-coated with Fibronectin (1:200 dilution, #F1141; Sigma-Aldrich, St. Louis, USA). Cells were cultured for five weeks in Gibco KnockOut DMEM supplemented with 2-mM L-glutamine, 5% (v/v) KnockOut Serum Replacement, 0.1-mM MEM Non-Essential Amino Acids, 5-µg/mL gentamycin, 0.1-mM β-mercaptoethanol, and 10-mM nicotinamide (all from Life Technologies, unless otherwise specified).

### Computational design and evaluation of targeting sgRNAs

sgRNAs were designed using online platforms BENCHLING (http://benchling.com) and CRISPOR (http://crispor.tefor.net/). In vitro validation of sgRNA efficacy and allele specificity was conducted using a fluorescence-based reporter assay in HEK293T cells, as described by Mashiko et al. [39]. Genomic fragments (300–500 bp) containing either the wildtype or mutant BEST1 alleles were cloned between the 5′ and 3′ enhanced green fluorescent protein (EGFP) fragments of the pCAG-EGxxFP plasmid (Addgene #50716; gift from Masahito Ikawa, MA, USA).

To express *Streptococcus pyogenes* Cas9 (SpCas9) and the sgRNA, the plasmid pU6-(BbsI) CBh-Cas9-T2A-mCherry (px330_mcherry, Addgene #64324, gift from Ralf Kuehn) was used, following the Zhang Lab General Cloning Protocol (https://www.addgene.org/crispr/zhang/). HEK293T cells were co-transfected with the target-containing pCAG-EGxxFP plasmid and pX330-mcherry. After 48 h, cells were reseeded into black 96-well plates and incubated for an additional 24 h. Nuclei were stained with Hoechst 33,342 (Sigma-Aldrich, St. Louis, USA), and fluorescence was measured 72 h post-transfection using a Tecan plate reader. EGFP signal was normalized to both mCherry expression and Hoechst fluorescence. Primer and sgRNA sequences are listed in Additional File 1: Table S1.

### Genome editing in hiPSCs

Genome editing in hiPSC cells was performed via electroporation of preassembled Cas9/sgRNA ribonucleoprotein (RNP) complexes. The sgRNA targeting the BEST1-I295del mutation was synthesized using the GeneArt Precision gRNA Synthesis Kit (Thermo Fisher Scientific, Waltham, USA). Briefly, the sgRNA template was generated by PCR amplification of two single-stranded oligonucleotides: 5’-*TAATACGACTCACTATAG*gagcagctcaacccctt-3’ (SZ_I295del_17nt-F) and 5’-*TTCTAGCTCTAAAAC*aaggggttgagctgctc-3’ (SZ_I295del_17nt-R), with the target sequence in bold and the T7 promoter sequence underlined. Following in vitro transcription and purification, 3 µg of sgRNA was complexed with 3 µg of recombinant GeneArt™ TrueCut™ *Streptococcus pyogenes* Cas9 nuclease (Thermo Fisher Scientific, Waltham, USA) for 8 min at room temperature under nuclease-free conditions to form the RNP complex. For genome editing, 800,000 hiPSCs were electroporated with the RNP complexes using the Amaxa Nucleofector and the Stemcell Nucleofector™ Kit VPH-5022 (Lonza, Basel, Switzerland), under program B-016, following the manufacturer’s instructions.

### Generation of clonal stem cell populations

Following RNP-mediated editing, hiPSCs were dissociated into single-cells and cultured in mTeSR1 medium (STEMCELL Technologies Germany GmbH, Cologne, Germany) to establish clonal populations. Individual clones were expanded and split into duplicates for downstream indel analysis.

gDNA was extracted from 50 to 100 hiPSC colonies and the BEST1 target region at p.(I295del) was amplified by PCR using primers flanking the Cas9 cleavage site. Amplicons were subcloned into the pGEM-T PCR vector (Promega, Madison, USA), and individual alleles were analyzed by Sanger sequencing. Clones harboring frameshift mutations and predicted premature stop codons were selected for further differentiation into RPE cells and subsequent phenotypic characterization.

### Genome editing in hiPSC-RPE cells

For genome editing in differentiated hiPSC-RPE cells, sgRNA and SpCas9 were delivered via plasmid transfection. Cells were transfected with the px330_mcherry plasmid (Addgene #64324) using Lipofectamine™ 3000 (Thermo Fisher Scientific), following the manufacturer’s instructions.

At 48 h post-transfection, mCherry-positive cells were isolated via fluorescence-activated cell sorting (FACS) at the Flow Cytometry Facility of the Regensburger Center of Interventional Immunology (RCI). gDNA was extracted from sorted cells for validation of genome editing efficiency and downstream molecular analyses.

### Determination of editing frequency

To assess genome editing efficiency at the on-target and predicted off-target loci in FACS-sorted hiPSC-RPEs, gDNA was extracted and subjected to PCR amplification using primer pairs flanking the target regions (Additional File 1: Table S1). PCR amplicons were subcloned into the PCR-vector pGEM-T Easy vector (Promega), and > 100 individual clones per transfection were analyzed by Sanger sequencing to quantify indel events (Additional File 2: Fig. S2).

### Genome-wide off-target analysis by whole genome sequencing (WGS)

WGS of FACS-sorted, mCherry-positive hiPSC-RPEs was performed using the NovaSeq X Plus platform (Illumina, Berlin, Germany) at an average coverage of 30x per sample. DNA input for library preparation was approximately 0.25 µg per sample. Next-generation sequencing data were processed using the CLC Biomedical Genomics Workbench (Qiagen), and reads were aligned to the human reference genome (GRCh38). Putative off-target sites were predicted using Cas-OFFinder (http://www.rgenome.net/cas-offinder/), applying the protospacer adjacent motif (PAM) 5′-NRG-3′ (R = A or G). Criteria for mismatch tolerance were as follows: up to 1 mismatch for 17-nt sgRNAs, and up to 3 mismatches for 20-nt sgRNAs. All predicted sites were manually reviewed using the Integrative Genomics Viewer (IGV) to confirm off-target activity.

### RNA isolation and reverse transcription (RT)

Total RNA was extracted from hiPSC-RPEs using the RNeasy Mini Kit (Qiagen, Hilden, Germany), including on-column DNase I treatment to eliminate gDNA contamination. First-strand cDNA was synthesized from 1 µg of total RNA using the RevertAid^™^ Reverse Transcriptase and Random Hexamer Primers (Thermo Fisher Scientific, Waltham, USA). RT-PCR was performed to assess the expression of hiPSC- and RPE-specific marker genes, as described [37]. Primer sequences are listed in Additional File 1: Table S4.

### SDS-page and Western blot analysis

Protein extraction, SDS-polyacrylamide gel electrophoresis (SDS-PAGE), and Western blot analysis were conducted as previously described [11, 20]. Briefly, protein samples were separated on 10% SDS-PAGE gels and transferred to Immobilon-FL membranes (LI-COR Bioscience, Bad Homburg, Germany). Primary and secondary antibody incubations were performed overnight at 4 °C. Protein signals were detected via near-infrared fluorescence using the Odyssey Fc Imaging System, and signal intensities were quantified using Image Studio software. Target protein levels were normalized to ß-actin as an internal control from the same blot.

### Immunofluorescence procedure and microscopy

Immunofluorescence staining of hiPSC-RPE cells was performed as previously described [13, 20]. Briefly, monolayers cultured on Transwell^®^ filters for six weeks were fixed with 4% paraformaldehyde in phosphate-buffered saline (PBS) for 10 min at room temperature. Cells were then permeabilized and blocked using PBS containing 0.3% Triton X-100 and 10% goat serum for 25 min. Primary antibody incubation (anti-BEST1) was carried out overnight at 4 °C, followed by incubation with fluorescently conjugated secondary antibodies under the same conditions. Stained cells were imaged using a confocal laser scanning microscope (FV3000 Fluoview; Olympus Life Sciences, Hamburg, Germany). Phase-contrast images were acquired using an inverted microscope (Nikon Eclipse TE2000-S; Nikon Instruments Europe BV, Netherlands).

### YFP-halide transport assay

The halide-sensitive yellow fluorescent protein (YFP) transport assay was conducted as described [13]. Lentiviral vectors were produced by co-transfecting HEK293T cells with the pLJM1 vector encoding yellow fluorescence protein YFP (H148Q/I152L) along with packaging plasmids pMD2.G and psPAX2, using the calcium phosphate transfection method. hiPSC-RPE cells were transduced with lentiviral particles and cultured for three weeks on six-well Transwell^®^ inserts. Cells were then reseeded onto black 96-well microtiter plates and cultured for an additional week. For the assay, cells were incubated in an iodide (I^-^)-containing buffer (40 mM) supplemented with the calcium ionophore A23187 (5 µM; #C7522, Sigma-Aldrich, St. Louis, USA). Quenching of YFP fluorescence, indicative of iodide influx, was measured using a Spark^®^ Multimode Microplate Reader (Tecan Group Ltd., Männedorf, Switzerland). After fluorescence quenching, the iodide solution was replaced with an equimolar chloride (Cl^-^)-containing buffer. The recovery of YFP fluorescence was monitored over a 5-minute period at 60-second intervals.

## Results

### Computational design of targeting sgRNAs

Our cohort of 197 unrelated index patients with BD was analyzed at the DNA Diagnostics Unit of the Institute of Human Genetics in Regensburg, Germany. Three well-established mutations in the *BEST1* gene (NM_004183.4) were found to be the most frequent: p.(A243V) (20%; *n* = 38), p.(I295del) (15%; *n* = 29) and p.(R218C) (6%; *n* = 11). All three mono-allelic mutations have previously been reported to cause either complete or partial loss of the BEST1 channel activity [13, 15, 29]. Consequently, for the CRISPR/Cas9-based suppression of the mutant allele, the development of only three mutation-specific sgRNAs could potentially enable treatment for approximately 40% of individuals in our and possibly other BD cohorts worldwide.

**Fig. 1:**
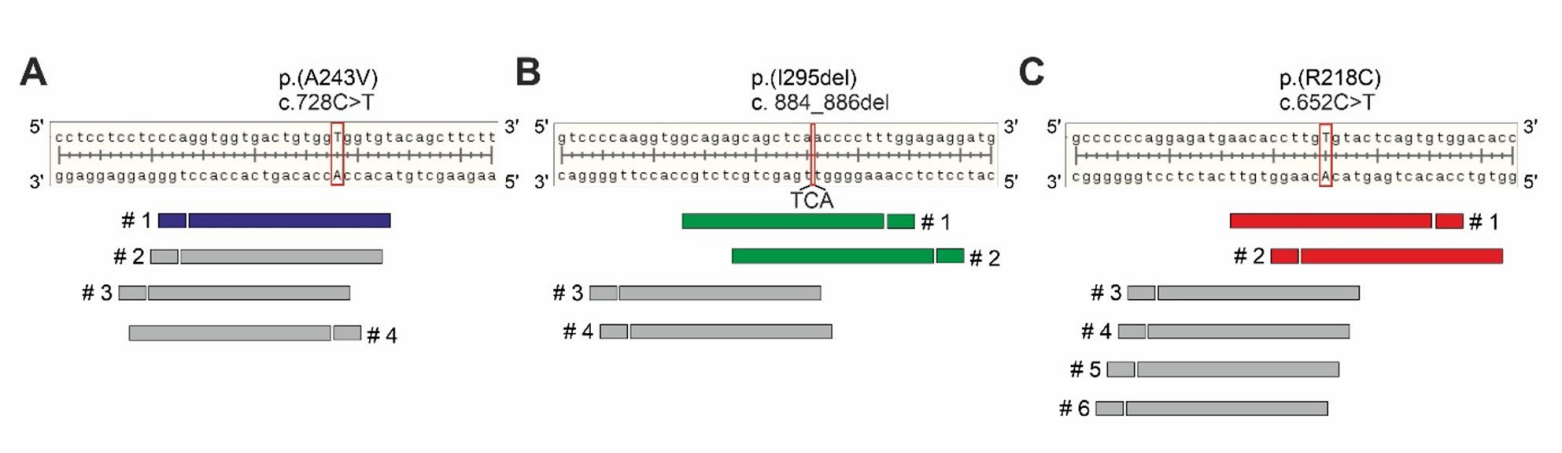
Computational design of allele-specific sgRNAs. High-quality sgRNAs recognizing the protospacer adjacent motif (PAM) sequence 5′-NGG-3′ were designed using the prediction tools BENCHLING (benchling.com) and CRISPOR (crispor.tefor.net). The design targeted three pathogenic variants in the *BEST1* gene: (**A**) a C > T substitution at nt position c.728 in exon 7 (p.(A243V)) (NC_000011.10:g.61958159 C > T), (**B**) a 3 bp deletion (TCA) spanning positions c.884–886 in exon 8 (p.(I295del)) (NC_000011.10:61959510:TCATCA: TCA), and (**C**) a C > T substitution at position c.652 in exon 6 (p.(R218C)) (NC_000011.10:g.61957402 C > T). Guide sequences were configured to be 20-nt in length. Candidate sgRNAs were prioritized based on scoring metrics provided by the prediction platforms, and selected candidates (highlighted by color) were chosen for subsequent in vitro validation

To design allele-specific sgRNAs computationally, we focused on the SpCas9 system, which recognizes the PAM sequence 5`-NGG-3`, as it remains the most widely used endonuclease enzyme in genome engineering. We employed the software tools BENCHLING and CRISPOR to predict high quality 20-nt sgRNA sequences with strong on-target activity and minimized off-target potential (each with prediction scores > 50%). By ensuring that the mutated base was located either within the PAM sequence or the 20-nt region adjacent to it, we identified four candidate sgRNAs for p.(A243V) (Fig. 1A), four for p.(I295del) (Fig. 1B), and six for p.(R218C) (Fig. 1C).

Out of the 14 candidate sgRNAs, four high-quality guides were identified: sgRNA I295del sgRNAs #1 and #2 and R218C sgRNAs #1 and #2 (Fig. 1; Table 1). Selection was based on two primary criteria: (i) on- and off-target scores ranging from 43 to 75, indicating acceptable predicted specificity and efficiency, and (ii) inclusion of the nucleotide alteration within the seed region of the sgRNA, as mismatches beyond the first 8–12 nt are known to diminish targeting specificity [40]. None of the sgRNAs targeting p.(A243V) fulfilled both criteria. As a result, A243V sgRNA #1 was chosen for further analysis, due to the advantageous positioning of the mutated nucleotide proximal to the PAM site, which is critical for efficient recognition by SpCas9. In contrast, A243V sgRNA #4 was excluded, as the mutation is embedded within the variable “N” position of the PAM motif (5′-NGG-3′), rendering the mutant and wildtype alleles indistinguishable and compromising allelic specificity.

**Table 1:**
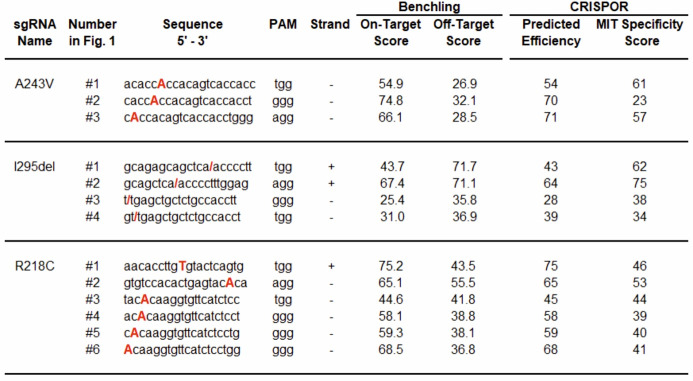
On- and off-target scores of predicted sgRNAs*

*On-target activity and potential off-target effects were predicted for each sgRNA using SpCas9-specific algorithms. Higher on-target and predicted efficiency scores correlate with an increased likelihood of successful cleavage at the intended genomic site [41]. Off-target risk was assessed using the MIT specificity score, which reflects the uniqueness of a guide sequence within the genome. A higher specificity score indicates reduced likelihood of off-target cleavage events [40]. Both scoring systems yield values ranging from 0 to 100, with higher scores representing better performance. Where possible, scores > 50 are recommended to ensure a balance between efficient editing and minimal off-target activity.

Based on their favorable on-target and off-target profiles five sgRNAs were selected for further in vitro validation: A243V sgRNA #1 (Fig. 1A, blue bar), I295del sgRNA #1 and #2 (Fig. 1B, green bars), and R218C sgRNA #1 and #2 (Fig. 1C, red bars) testing.

### In vitro evaluation of sgRNAs

**Fig. 2:**
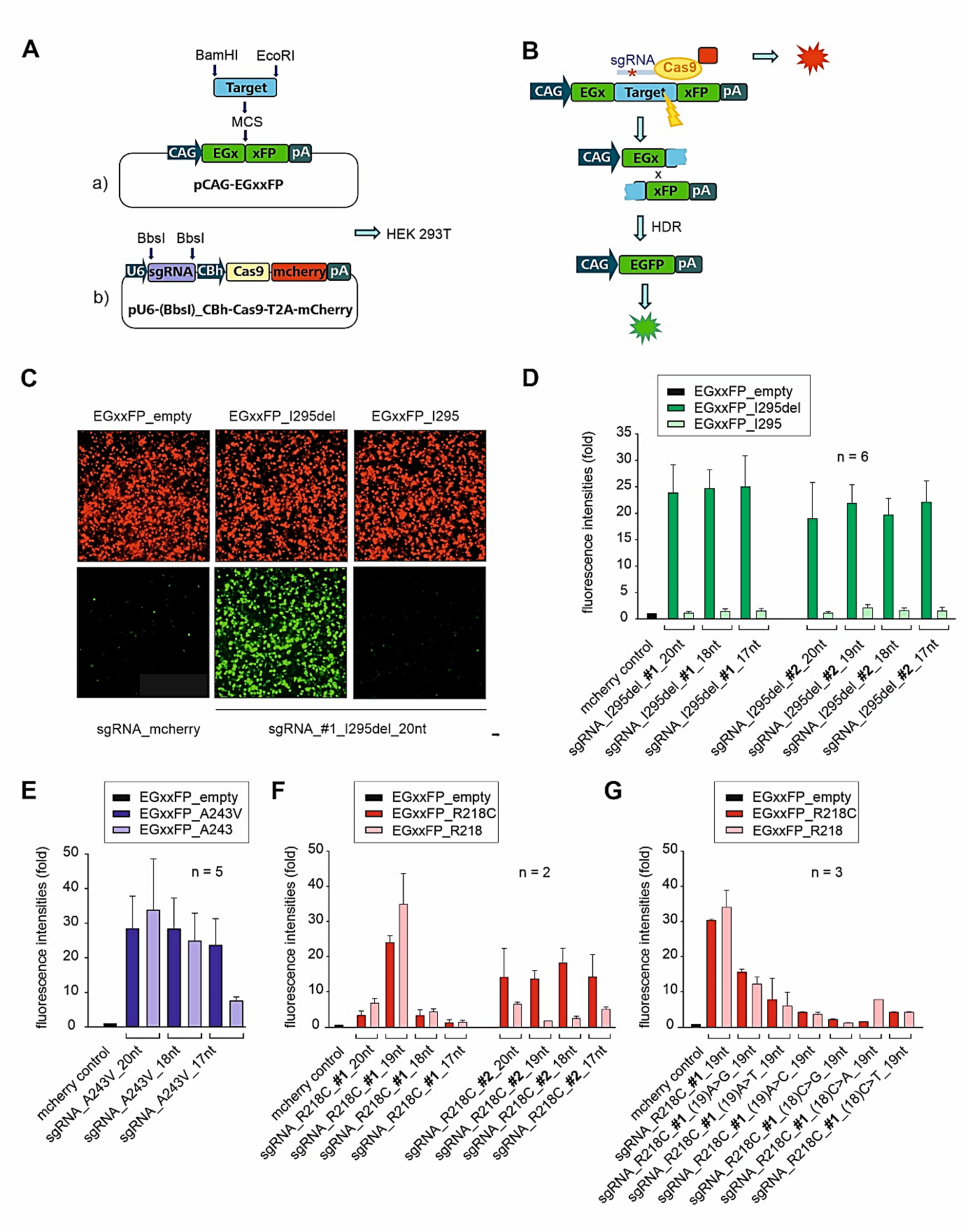
Evaluation of sgRNA efficacy and specificity in HEK293T cells. **A** Schematic view of the fluorescence-based reporter assay. HEK293T cells were co-transfected with (a) the pCAG-EGxxFP reporter plasmid, containing a multicloning site (MCS) inserted between EGFP fragments sharing 482 bp under the control of a chicken beta-actin (CAG) promoter, and b) the pU6-(BbsI)_CBh-Cas9-T2A-mCherry (pX330-mCherry) plasmid, which allows directional cloning of sgRNA oligos into BbsI sites under a U6 promoter and co-expresses SpCas9 and mCherry (red fluorescence) driven by a chicken β-actin hybrid (CBA) promoter. **B** A 500 bp genomic fragment containing the sgRNA target sequence (“target”) was inserted between the two split EGFP fragments of the pCAG-EGxxFP plasmid. The resulting target plasmid was co-transfected with the pX330 plasmid expressing the respective sgRNA and SpCas9 into HEK293T cells. Upon Cas9-mediated cleavage directed by the sgRNA, homology-directed repair (HDR) reconstitutes the EGFP expression cassette, leading to restoration of green fluorescence. **C** Representative immunofluorescence imaging of HEK293T cells transfected with pCAG-EGxxFP (empty vector) (left), pCAG-EG_on-target_I295del_FP + sgRNA_I295del_#1_20nt (middle) or pCAG-EG_non-target_I295_FP + I295del_#1_20nt (right); mCherry serves as a transfection control, expressed from the mCherry-containing px330-sgRNA-SpCas9-vector; (**D-G**) Quantification of EGFP fluorescence intensities relative to the empty pCAG-EGxxFP vector by plate reader measurements. HEK293T cells were transfected with pCAG-EG_on-target_FP + sgRNA_X or pCAG-EG_non-target_FP + sgRNA_X after sgRNA truncation (**D-F**) and additional sequence modifications (**G**)

A major challenge in applying the CRISPR/SpCas9-sgRNA system lies in balancing editing efficiency and specificity, as SpCas9 can tolerate up to six base mismatches within the protospacer or even a single base change in the PAM sequence [42]. To enhance target discrimination, we generated truncated sgRNAs (17-19nt) for each of the five selected full-length (20-nt) sgRNAs, based on evidence suggesting that shortening the guide sequence can improve specificity [43]. To evaluate editing efficiency and allele specificity in vitro, we first employed a fluorescence-based reporter assay in HEK293T cells (Fig. 2) [39].

For each mutation, genomic fragments of 250–500 bp containing either the on-target or the non-target (allelic wildtype) sequence were cloned into the EGxxFP reporter plasmid (pCAG-EGxxFP) (Fig. 2A), where they were inserted between two non-fluorescent EGFP fragments [39]. The resulting target plasmids were co-transfected with SpCas9-expressing px330-mCherry plasmids harboring the respective sgRNAs [44]. Upon cleavage of the target site, homology-directed repair restores the EGFP reading frame (Fig. 2B and C), and EGFP fluorescence was quantified 72 h post-transfection (Fig. 2D-G).

Plate reader analysis confirmed high-editing efficacy at the I295del sgRNA #1 and #2 and A243V sgRNA #1, each showing a ~ 25- to 45-fold increase in fluorescence compared to baseline (Fig. 2D-E). In contrast, R218C sgRNAs _#1 and #2 displayed substantially lower efficacy, with increases of approximately 2-8-fold and 14-24-fold, respectively (Fig. 2F). An exception was R218C_#1_19nt, which showed a ~ 38-fold fluorescence, indicating strong cleavage activity. However, this truncated guide failed to discriminate between the mutant and wildtype alleles, highlighting a loss of allele specificity.

To address this, we introduced additional base mismatches at positions 18 and 19 of R218C_#1_19nt to further reduce binding affinity to the non-target allele. This introduced a new mismatch with the on-target sequence and a second mismatch with the off-target allele. However, all tested variants led to a marked reduction in editing efficacy, as compared to the original 19-nt version (Fig. 2G). Consequently, R218C sgRNA #1 was excluded from further analysis.

Similarly, A243V sgRNAs (20-nt and 18-nt) exhibited low allele specificity, allowing substantial cleavage of the off-target allele. This indicates that a single mismatch outside the seed region was insufficient to prevent SpCas9 activity. Notably, truncation to 17-nt significantly improved allele specificity for the A243V sgRNA (Fig. 2E), suggesting that guide shortening can enhance discrimination in specific sequence contexts.

Taken together, our findings underscore that allele specificity is highly dependent on the nucleotide composition and structure of each sgRNA. Therefore, empirical testing of multiple guide lengths and sequence variants is essential to optimize specificity without concomitant loss of efficacy.

### Evaluation of sgRNAs in hiPSC-RPE cell lines derived from BD patients

To validate the findings from the initial HEK293T fluorescence-based assay in a more physiologically relevant model, we evaluated sgRNA activity in hiPSC-RPE cell lines. These lines, previously established in our laboratory [13], are heterozygous for *BEST1* mutations p.(A243V), p.(I295del), and p.(R218C), and thus represent appropriate cellular models of BD.

CRISPR/SpCas9 components were introduced via electroporation into the respective mutant hiPSC-RPE lines. mCherry-positive cells were collected 72 h post-transfection to assess editing outcomes. To evaluate editing events, both target and non-target alleles were amplified using oligonucleotide primers flanking the respective *BEST1* mutation sites. Amplicons from approximately 50–100 single clones were generated from 2 to 6 independent transfection experiments and subjected to Sanger sequencing to determine allele-specific insertion and deletion (indel) frequencies (Additional File 2: Fig. S2).

Among the seven sgRNAs tested (A243V_17nt, R218C_#2_20nt, R218C_#2_17nt, I295del_#1_20nt, I295del_#1_17nt, I295del_#2_20nt and I295del_#2_17nt), no detectable DSBs were observed at any of the non-target (wildtype) alleles, indicating high allele specificity across all guides (Table 2). However, only the four I295del-targeting sgRNAs (I295del_#1_20nt, I295del_#1_17nt, I295del_#2_20nt and I295del_#2_17nt) achieved robust on-target editing, with indel frequencies ranging from 23 to 58% of clones tested. In contrast, sgRNAs targeting R218C and A243V exhibited substantially lower cleavage efficiencies, yielding indel frequencies of approximately 4% and 10%, respectively. As anticipated, approximately two-thirds of all edited alleles resulted in frameshift mutations, suggesting a high probability of nonsense-mediated mRNA decay (NMD) and effective knockout of the mutant *BEST1* allele. Of note, cleavage efficacies in hiPSC-RPE cells varied substantially, and in many cases, did not correlate with results obtained in HEK293T co-transfection assays. This discrepancy highlights the unpredictability of sgRNA performance across different cellular contexts and underscores the critical importance of empirically testing each sgRNA in the relevant disease model prior to therapeutic application. An overview of all sgRNAs evaluated in hiPSC-RPE cells and their respective performance metrics is provided in Table 2.

**Table 2:** Quantification of indel frequencies of Sanger-sequenced amplicons from CRISPR/SpCas9-treated hiPSC-RPE cells*

Clones carrying allele	sgRNA (no. of transfections)	no. of clones	CRISPR/Cas9-positive clones	Frequency (%)
wildtype	A243V_17nt (6)	189	0	0
mutant		172	17	10
wildtype	I295del_#1_20nt (3)	91	0	0
mutant		29	12	41
wildtype	I295del_#1_17nt (5)	118	0	0
mutant		73	42	58
wildtype	I295del_#2_20nt (2)	46	0	0
mutant		39	10	26
wildtype	I295del_#2_17nt (3)	79	0	0
mutant		92	29	23
wildtype	R218C_#2_20nt (4)	115	0	0
mutant		105	8	8
wildtype	R218C_#2_17nt (3)	116	0	0
mutant		114	4	4

*Sanger sequencing of approximately 50–100 single clones from 2–6 independent transfection rounds provided the sequence data

### Off-target analysis

To evaluate potential off-target effects of CRISPR/SpCas9 editing, we conducted deep sequencing of mCherry-positive hiPSC-RPE cells treated with the following sgRNAs: A243V_17nt, I295del_#1_20nt, I295del_#1_17nt, I295del_#2_20nt, I295del_#2_17nt, R218C_#2_20nt and R218C_#2_17nt. For sgRNA A243V_17nt, analysis of the top 17 predicted off-target sites revealed detectable editing at two loci: one located in a non-coding region on chromosome 8 (chr8:47195791–47195817 (GRCh37/hg19)) and another within the coding region of the *TAGLN2* gene on chromosome 1 (chr1:159921809–159921812 (GRCh37/hg19)) with editing frequencies of approximately 6% and 3%, respectively. In the case of sgRNA R218C_#2_20nt, we observed low-level editing at a previously reported off-target site within a non-coding region of chromosome 7 (chr7:10314110–10314114 (GRCh37/hg19)). Importantly, this off-target event was not detected when using the truncated variant, R218C_#2_17nt, suggesting improved specificity.

All four sgRNAs targeting the I295del mutation exhibited no detectable off-target activity at their respective predicted sites, as confirmed by deep sequencing (Additional File 3: Table S5). Based on these findings, sgRNA I295del_#1_17nt was selected for subsequent experiments to assess the impact of allele-specific genome editing on molecular phenotypes and BEST1 channel function in RPE cell lines derived from patients with BD.

### Effect of the I295del Mutation on BEST1 localization, protein stability, and anion permeability in hiPSC-RPE cells

To evaluate the pathogenic impact of the heterozygous I295del mutation in *BEST1*, we utilized hiPSC-RPE cells from two unrelated BD patients (AP_+/I295del_ and MD_+/I295del_) alongside cells from two healthy donors (MK_control_ and AM_control_). These lines were selected for their suitability in investigating allele-specific CRISPR/Cas9 genome editing as a therapeutic strategy for BD, as previously described by Nachtigal et al. [13]).

**Fig. 3:**
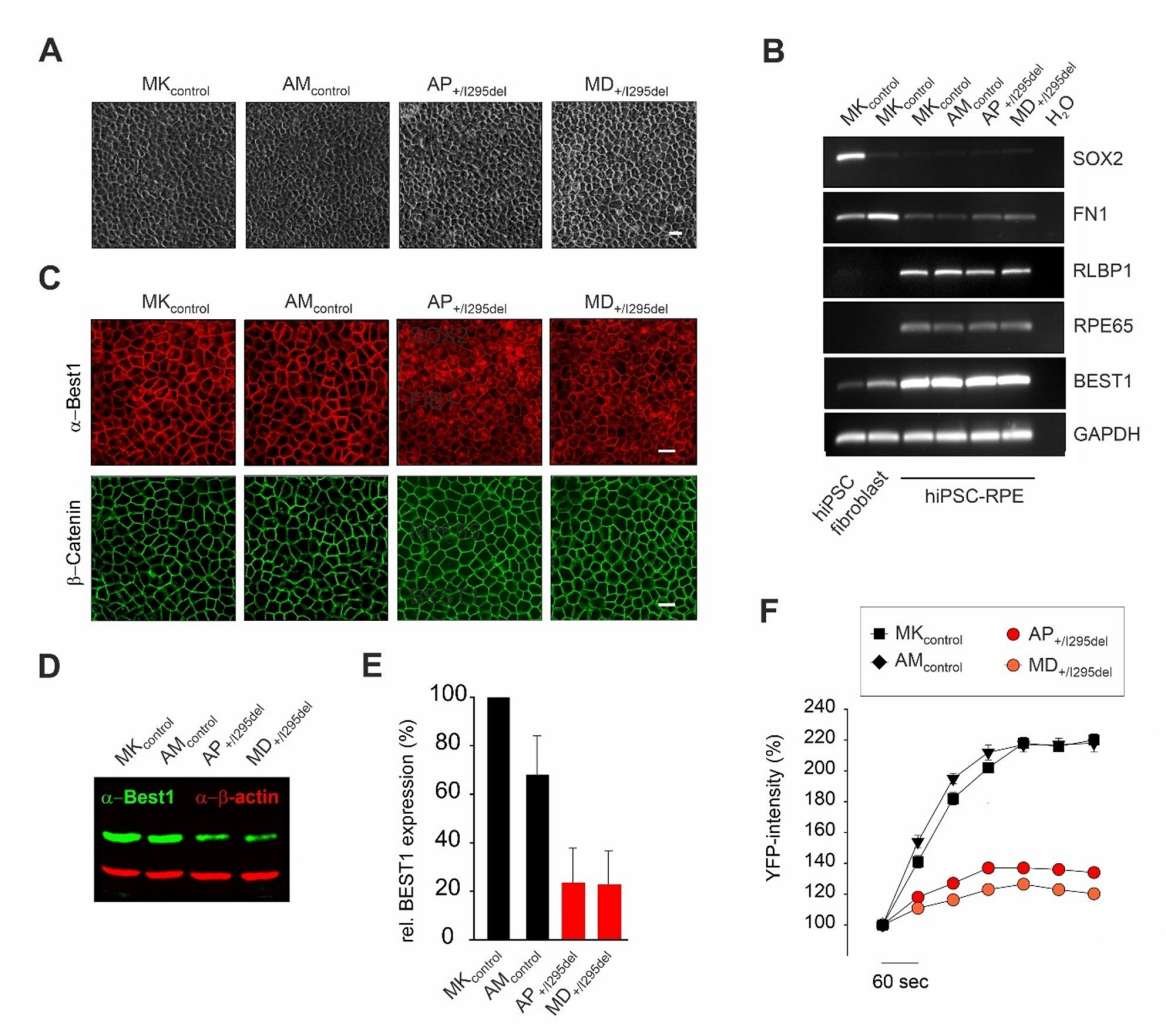
Characterization of hiPSC-RPE cell lines derived from BD patients and healthy controls. **A** After six weeks of maturation on Transwell^®^ inserts, all four hiPSC-RPE lines exhibited the characteristic pigmented, hexagonal morphology typical of mature RPE cells. **B** Quantitative analysis of mRNA expression levels for key RPE-specific marker genes (*BEST1*, *RPE65* and *RLBP1*), the pluripotency marker *SOX2*, and fibronectin 1 (*FN1*) was performed. Expression profiles of the hiPSC-RPE lines MK_control_, AM_control_, AP_+/I295del_ and MD_+/I295del_ were compared to the parental fibroblasts and undifferentiated hiPSC cells of MK_control_, confirming successful differentiation and RPE identity. **C** Confocal immunofluorescence staining of indicated control and BD hiPSC-RPE cells was performed using antibodies against BEST1 (red) and β-catenin (CTNNB1) (green). In healthy control lines, BEST1 localized prominently to the basolateral plasma membrane, co-localizing with CTNNB1, a known marker of basolateral polarity. In contrast, hiPSC-RPE cells from BD patients (AP_⁺/I295del_ and MD_⁺/I295del_) displayed a redistribution of BEST1 to intracellular compartments, indicating altered protein trafficking. Scale bar: 20 μm. **D** Representative Western blot images of whole-cell lysates from control and BD hiPSC-RPE lines, probed with an antibody against human BEST1. Beta-actin was used as a loading control. **E** Densitometric quantification of BEST1 protein levels from (D) revealed a marked reduction in expression in both BD-associated lines (AP_+/I295del_ and MD_+/I295del_) compared to the healthy control lines. **F** Functional assessment of anion permeability using the YFP-halide transport assay. hiPSC-RPE cells were loaded with the YFP-based halide sensor and first incubated with 40 mM sodium iodide, followed by replacement with sodium chloride. The resulting efflux of iodide and influx of chloride ions induced an increase in YFP fluorescence intensity, reflecting BEST1 channel activity. Healthy control lines exhibited rapid and robust increases in fluorescence, whereas the BD patient-derived lines showed minimal signal change, consistent with impaired chloride conductance. Data presented as mean ± SD from independent biological replicates, each data point represents the mean of six technical replicates

Human dermal fibroblasts were reprogrammed into hiPSCs and RT-PCR analysis confirmed expression of stem cell–specific markers compared with a dermal fibroblast control line (Additional File 2: Fig. S1A). HiPSCs were then differentiated into RPE cells following the protocol established by Brandl et al. [37]. After six weeks of maturation on Transwell^®^ inserts, all four hiPSC-RPE lines exhibited typical pigmented hexagonal morphology (Fig. 3A) and robust expression of characteristic RPE marker genes (Fig. 3B).

#### BEST1 localization and protein stability

To investigate how the I295del mutation affects BEST1 protein behavior, we assessed its localization and expression via confocal microscopy and quantitative Western blotting. Immunofluorescence analysis revealed that in hiPSC-RPE cells from healthy controls, BEST1 was prominently localized to the basolateral plasma membrane. In contrast, BD-associated cells displayed markedly weaker membrane localization, with a substantial proportion of BEST1 retained intracellularly (Fig. 3C). Quantitative Western blot analysis of whole-cell extracts further demonstrated a significant reduction in total BEST1 protein expression in AP_⁺/I295del_ and MD_⁺/I295del_ cells, reaching only ~ 23% of control levels at steady-state (Fig. 3D and E), indicating reduced protein stability or increased degradation associated with the mutation.

#### Functional impact on anion permeability

To assess the functional consequences of the I295del mutation, we employed the YFP-halide transport assay, a well-established method for measuring anion flux through BEST1 channels [13, 45]. All four hiPSC-RPE lines were transduced with the halide-sensitive reporter YFP_(H148Q/I152L)_, and fluorescence intensity was measured over a five-minute period to monitor anion efflux mediated by BEST1 channels. In healthy control hiPSC-RPEs, a rapid and robust increase in fluorescence (up to ~ 220%) was observed, consistent with normal BEST1-mediated chloride transport. In contrast, both BD-associated cell lines exhibited minimal fluorescence changes, indicating severely impaired anion permeability likely due to the I295del mutation (Fig. 3F).

### CRISPR/SpCas9 gene editing in hiPSCs from BD patients

Delivery of the Cas9 protein as an RNP complex with sgRNA offers key advantages over plasmid-based approaches. RNP complexes initiate genome editing immediately upon cellular entry and are rapidly degraded, thereby reducing the duration of Cas9 activity and minimizing off-target effects [46].

To apply this strategy, hiPSCs derived from two BD patients carrying the heterozygous *BEST1*-I295del mutation were electroporated with SpCas9/sgRNA_I295del_17nt_ RNPs. Following transfection, single cell clones were expanded and screened for successful editing by analyzing the occurrence of DSBs at the target locus. From each patient line, two genome-edited pluripotent clones with confirmed pluripotency (see Additional File 2: Fig. S1B) were selected (AP_crispr#16_ and AP_crispr#19_ from the AP_+/I295del_line), and MD_crispr#2_ and MD_crispr#22_ from the MD_⁺/I295del_line). These clones harbored confirmed DSB-induced frameshift mutations resulting in premature stop codons, as validated by sequencing (Additional File 2: Fig. S3). These edited clones were subsequently directed through RPE differentiation to generate CRISPR/SpCas9-edited hiPSC-RPE lines for further phenotypic and functional analyses.

To control for potential clonal artefacts arising from the single-cell cloning process, we additionally selected two single cell derived populations (MD_+/I295del__#1 and MD_+/I295del__#3) from the RNP-electroporated MD_⁺/I295del_ line that exhibited no detectable genome editing (i.e., no Cas9-induced cleavage). These control clones are expected to be phenotypically indistinguishable from the parental hiPSC-RPE line MD_⁺/I295del_ and serve as important references for downstream comparisons (Additional File 2: Fig. S3).

### Loss of the mutant transcript in edited hiPSC-RPE cells

To assess the impact of CRISPR/SpCas9-mediated editing on *BEST1* expression, we first performed Sanger sequencing to compare both gDNA and cDNA from the four SpCas9-edited hiPSC-RPE lines with their corresponding untreated patient-derived cell lines.

**Fig. 4:**
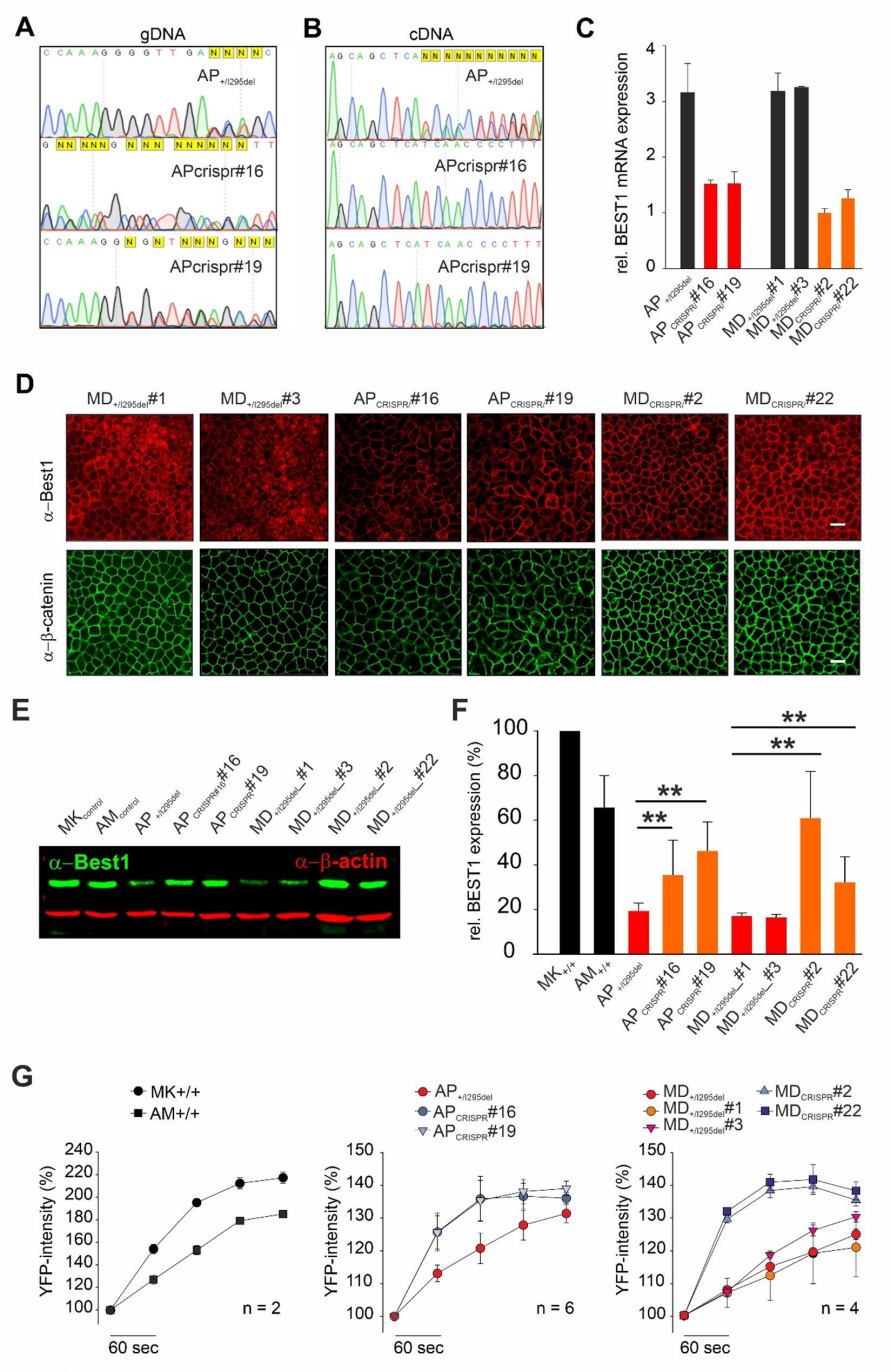
Analysis of BEST1 mRNA and protein expression, protein localization and channel function in untreated and CRISPR/SpCas9-edited hiPSC-RPE cell lines following 6 weeks of maturation on Transwell^®^ inserts. Sanger sequencing of the *BEST1*-I295del locus in (**A**) genomic DNA (gDNA) and (**B**) complementary DNA (cDNA) from the indicated untreated and CRISPR/SpCas9-edited hiPSC-RPE lines. Sequences were visualized using SnapGene software, illustrating the presence or absence of the mutant transcript. **C** Quantitative RT-PCR analysis of *BEST1* mRNA expression in untreated and CRISPR/SpCas9-edited hiPSC-RPE cell lines. Total RNA was extracted, reverse transcribed, and measured in triplicates across three biological replicates. Data were normalized to *HPRT1* expression. Color coding: Black = untreated lines; red = CRISPR-edited clones derived from parental line AP_+/I295del_; orange = CRISPR-edited clones derived from parental cell line MD_+/I295del_. **D** Confocal immunofluorescence staining of clonal hiPSC-RPE lines using antibodies against human BEST1 (red). ß-catenin (green) to assess protein localization. Scale bar = 20 μm. **E** Representative Western blot of whole-cell lysates from untreated and CRISPR/SpCas9-edited lines, probed with antibodies against human BEST1 (green) and anti-ACTB as loading control (red). **F** Quantification of BEST1 protein levels from panel (E), normalized to ACTB and expressed relative to healthy control line MK+/+. Data represent mean ± SD from two independent experiments (each with two technical replicates; *n* = 4 total). Statistical significance was assessed using a two-tailed paired Student’s t test; **, *P* < 0.01, relative to untreated lines AP_+/I295del_, MD_+/I295del_#1, and MD_+/I295del_#3. **G** YFP fluorescence intensity in the halide transport assay for the indicated lines, used to assess BEST1-mediated anion channel function. Data presented mean ± SD of independent measurements, with each individual value calculated as the mean of six technical replicates

At the genomic level, all lines, including edited and unedited, exhibited overlapping sequencing traces, indicative of the presence of both wildtype and mutant alleles, the latter characterized by a 3 bp deletion (Fig. 4A and Additional File 2: Fig. S4 left). In contrast, analysis of *BEST1* expression at the transcript level revealed a clear distinction between treated and untreated samples. In the untreated patient cell lines (AP_⁺/I295del_, MD_+/I295del__#1 and MD_+/I295del__#3), cDNA sequencing showed heterozygous expression, with overlapping peaks corresponding to both wildtype and mutant transcripts at the on-target site (Fig. 4A and Additional File 2: Fig. S2, right). However, in all four SpCas9-edited hiPSC-RPE lines, only wildtype cDNA sequences were detected. The absence of mutant transcripts strongly supports the occurrence of NMD, likely triggered by frameshift-induced premature termination codons introduced by the genome editing. These results were corroborated by quantitative reverse transcriptase PCR, which showed an approximate 50% reduction in total *BEST1* mRNA levels in edited samples compared to their unedited counterparts (Fig. 4B). This decrease is consistent with selective degradation of the mutant transcript following successful allele-specific editing.

### Allele-specific disruption rescues the I295del phenotype in edited hiPSC-RPE cells

To assess the therapeutic impact of allele-specific editing, we analyzed BEST1 localization, protein expression and anion transport function in the SpCas9-edited hiPSC-RPE lines. The two untreated clonal control lines (MD_+/I295del__#1 and MD_+/I295del__#3) exhibited a pronounced mislocalization of BEST1 (Fig. 4D), consistent with the phenotype observed in the parental MD_+/I295del_ cell line (Fig. 3C). In contrast, all four edited SpCas9-edited hiPSC-RPE clones (AP_crispr#16_, AP_crispr#19_, MD_crispr#2_ and MD_crispr#22_) demonstrated robust basolateral membrane localization of BEST1, reflecting the expression of the wildtype allele only. Western blot analysis further supported these findings, showing a marked increase in BEST1 protein levels in all four edited lines. Notably, expression levels in the edited cells reached approximately 45% of those in healthy controls (MK_control_) (35 ± 16%, 46 ± 13%, 61 ± 21%, and 32 ± 12%, for AP_crispr#16_, AP_crispr#19_, MD_crispr#2_ and MD_crispr#22,_ respectively), indicating substantial restoration of protein stability and abundance (Fig. 4E and F). Importantly, this corresponds to a two-fold increase compared to the untreated mutant cell lines, which show only ~ 23% of healthy control protein levels (Fig. 3E), despite a ~ 50% reduction of BEST1 transcript abundance (Fig. 4C). This finding strongly supports a dominant-negative effect of the mutant allele, where removal of the pathogenic variant leads to marked recovery of protein stability and overall protein output. To evaluate functional recovery, we performed YFP-based halide transport assays to monitor BEST1-mediated anion permeability. The edited hiPSC-RPE clones (AP_crispr#16_, AP_crispr#19_, MD_crispr#2_ and MD_crispr#22_) exhibited a significant increase in YFP fluorescence, reflecting restored anion transport activity. This functional rescue contrasted sharply with the impaired anion transport observed in the untreated patient-derived lines (AP_+/I295del_ and MD_+/I295del_) and the two unedited clones (MD_+/I295del__#1 and MD_+/I295del__#3) (Fig. 4G).

Taking together, molecular and functional data demonstrate that allele-specific disruption of the mutant I295del allele via SpCas9-mediated genome editing rescues the disease phenotype in hiPSC-RPEs. These findings indicate that eliminating the dominant-negative effect of the mutant allele is more critical than restoring full *BEST1* dosage, reinforcing the potential of allele-specific gene editing as a therapeutic strategy for BD.

## Discussion

In this study, we systematically tested CRISPR/Cas9 sgRNAs targeting three common *BEST1* mutations (p.(A243V), p.(R218C), and p.(I295del)) for on-target editing efficiency, allele specificity, and genome-wide off-target effects. Using a fluorescence reporter assay in HEK293T cells and patient-derived hiPSC-RPE cells, we found that sgRNA sequence composition is the primary determinant of editing efficiency, whereas allele specificity and off-target effects can be refined by modifying guide length and sequence. We further showed that allele-specific CRISPR editing can restore BEST1 function in patient hiPSC-RPE cells by selectively inactivating the mutant allele while preserving the wildtype allele. In particular, CRISPR targeting of the dominant-negative I295del mutation in two BD patient-derived lines rescued key aspects of BEST1 expression, localization and anion transport function.

The *BEST1* mutation p.(A243V) is frequently associated with later-onset, and milder disease symptoms [47–50], suggesting a broader therapeutic window. In our study, an sgRNA targeting p.(A243V) showed high editing activity in HEK293T cells, consistent with strong predicted on-target scores (> 50) from two algorithms. However, the target nucleotide lies 15 bp upstream of the PAM site, an sgRNA position where mismatches are often tolerated [40, 42], limiting allele discrimination and risking wildtype allele cleavage. Truncating the guide to 17-nt improved specificity and retained high efficiency in HEK293T cells, but editing in patient hiPSC-RPE cells remained low (~ 10%), underscoring the limitations of simplified reporter assays in predicting outcomes in disease-relevant cells. Whole-genome sequencing confirmed substantial off-target editing at two genomic loci for the 17-nt guide, which likely sequestered Cas9 and reduced on-target activity. Consistently, this guide’s low specificity score (< 50) correlated with its diminished on-target performance in hiPSC-RPE cells.

The p.(R218C) alteration is another prevalent BD mutation, with allele frequencies of ~ 26% in Italian BD patients [51] and ~ 18% in Chinese patients [52], and typically causes the classic BD phenotype [47]. We designed two high-scoring sgRNAs for p.(R218C), but adjusting guide length and composition failed to improve allele specificity for one of them (sgRNA_R218C_#1). The second sgRNA achieved better allele discrimination, yet indel frequencies remained low in hiPSC-RPE cells for all p.(R218C) guides, mirroring the modest activity observed in HEK293T cells. Notably, our best p.(R218C) sgRNA (~ 8% editing in hiPSC-RPE cells) was much less efficient than reported by Sinha et al. (~ 66% editing with the same guide) [29]. This discrepancy likely stems from different methodologies. While Sinha et al. assessed editing via deep sequencing two weeks after lentiviral transduction, we measured double strand breaks 72 h after transient transfection. Moreover, it is expected that approximately one-third of edited cells will carry in-frame modifications at the target locus, which are unlikely to trigger transcript degradation and therefore may not result in functional correction. Importantly, such contrasts also highlight that delivery strategies will play a decisive role in translating allele-specific editing approaches into clinical applications, as delivery efficiency strongly influences both on-target activity and therapeutic feasibility. Currently, AAV-mediated delivery is considered the gold standard for ocular gene therapy [53], whereas lentiviral approaches may achieve higher transduction efficiency but raise additional safety concerns [54]. Non-viral delivery methods, including liposome- or nanoparticle-based systems, are also under investigation and may offer alternative routes for safe and efficient gene editing in the eye [55, 56].

The sgRNAs targeting the p.(I295del) mutation exhibited high on-target activity and robust allele specificity, owing to a 3 bp mismatch compared to the wildtype sequence. Whole-genome sequencing detected no off-target events for these guides. Using a truncated 17-nt sgRNA, we efficiently and selectively inactivated the mutant p.(I295del) allele in hiPSC-RPE cells, which improved BEST1 protein levels and partially restored function in two independent patient lines. The CRISPR-induced frameshift in the mutant allele introduced a premature stop codon predicted to trigger NMD. In our gene-edited p.(I295del) clones, the mutant *BEST1* mRNA was indeed undetectable (only wildtype transcripts were present), confirming that NMD eliminated the mutant transcript and its deleterious product. NMD typically targets transcripts with premature stop codons located > 50–55 nt upstream of an exon–exon junction. Such mutations frequently result in recessive inheritance patterns, as seen for the autosomal recessive bestrophinopathy [57] and other diseases [58]. However, exceptions exist [59], underscoring that experimental confirmation of NMD activation is needed. In this study, we observed complete absence of the mutant *BEST1* transcript in all four gene-edited hiPSC-RPE lines. Only the wildtype transcript was detectable at the heterozygous p.(I295del) locus, indicating that the premature stop codon triggers NMD and effectively abolished the dominant-negative effect of the p.(I295del) allele.

BEST1 normally localizes to the basolateral membrane of RPE cells [2, 13, 29, 60], a feature recapitulated in cellular hiPSC-RPE models [13]. Proper membrane targeting is associated with high protein stability, with a biological half-life exceeding 24 h [20]. By contrast, the I295del mutation causes BEST1 mislocalization in the cytoplasm, rapid endo-lysosomal degradation, and impaired anion transport across the RPE membrane [12, 13]. This dysfunction is evidenced by reduced chloride currents and lower membrane permeability in halide transport assays [10, 14, 29, 50]. In line with these known effects, our p.(I295del) hiPSC-RPE models showed mislocalized, unstable BEST1 and compromised anion transport. Crucially, after allele-specific editing of p.(I295del), BEST1 properly localized to the plasma membrane and protein levels rose to ~ 50% versus normal. Functional assays confirmed improved anion (chloride) transport, albeit not to wildtype levels, consistent with the presence of a single functional *BEST1* allele. Notably, heterozygous carriers of recessive *BEST1* nonsense mutations are clinically asymptomatic [6], indicating that a partial reduction in BEST1 expression is likely tolerable.

Over two decades after BEST1 was identified as the gene mutated in BD [3, 4], there is still no approved therapy. Gene therapy efforts for retinal diseases have so far focused on recessive and X-linked conditions. For BEST1, adeno-associated virus (AAV)-mediated gene supplementation is a promising approach. In a canine model of autosomal recessive bestrophinopathy, AAV-BEST1 delivery corrected retinal detachments and increased BEST1 expression [61]. A similar augmentation strategy might benefit autosomal dominant BD by boosting the fraction of functional BEST1 subunits in the pentameric channel [15, 29]. However, in dominant disorders mutant protein would still be expressed, potentially undermining long-term efficacy. This challenge has spurred interest in allele-specific therapies. Antisense oligonucleotides (AONs) can selectively knock down mutant transcripts [62], but they require lifelong repeated administration. In contrast, CRISPR/Cas offers a one-time, permanent solution via allele-specific knockout or an “ablate-and-replace” strategy that combines mutant allele inactivation with gene supplementation [14, 29, 63]. Notably, CRISPR-based allele-specific editing has shown success in other dominant retinal diseases, such as certain models of autosomal dominant retinitis pigmentosa [64, 65], underscoring its translational potential.

Although allele-specific CRISPR/Cas9 editing was successfully applied to correct the p.(I295del) mutation, we were unable to identify suitable sgRNAs for the p.(A243V) and p.(R218C) variants using the same strategy. For p.(A243V), the use of first-generation high-fidelity Cas9 variants such as SpCas9-HF1 or eSpCas9 may represent a promising alternative, as their increased specificity could reduce off-target effects and thereby render the available sgRNA suitable for on-target editing. For p.(R218C), high-fidelity Cas9 could also be beneficial for sgRNA R218C_#1_19nt, which exhibited strong cleavage activity but insufficient specificity. Although these nucleases typically sacrifice some cutting efficiency [66], their improved fidelity may broaden the feasibility of allele-specific targeting. Moreover, Cas9 variants with expanded PAM compatibility, particularly the near–PAM-less SpRY nuclease [67], could further increase the number of accessible target sites and thus provide additional options for precise allele-specific editing.

Next-generation genome editing strategies, such as base editing (BE) and prime editing (PE), may provide viable alternatives, however, these approaches were not the focus of the present study. Notably, BE and PE have been shown to correct the *Rpe65* mutation in the rd12 mouse, a model of Leber congenital amaurosis, improving visual function [68, 69], and both approaches efficiently repaired pathogenic mutations in the rd10 mouse model of retinitis pigmentosa [70, 71]. Compared with standard CRISPR/Cas9, BE and PE offer the advantage of precise correction of point mutations without generating double-stranded DNA breaks, potentially reducing off-target effects. However, limitations include restricted targetable sequences for BE and lower efficiency or larger construct sizes for PE, which may impact delivery to certain cell types or tissues.

## Conclusions

Overall, our findings establish a proof-of-concept for CRISPR/Cas-based allele-specific editing as a therapeutic strategy in autosomal dominant diseases. This precision genome editing approach could benefit patients with dominant-negative or gain-of-function mutations by selectively eliminating pathogenic alleles. Successful clinical translation, however, will require thorough evaluation of off-target effects, long-term safety, reliable allele discrimination in vivo and a carefully designed delivery strategy.

## Supplementary Information

Additional file 1. Table S1-S4.

Additional file 2. Figures S1-S4.

Additional file 3. Table S5. Genome-wide off-target analysis in CRISPR/Cas9-edited patient-derived hiPSC-RPE cell lines.

## Data Availability

Data generated in this study are included in the manuscript and supporting files. WGS data for off-target analysis are not publicly available due to IRB restrictions but can be shared under controlled access upon reasonable request to the corresponding author, in accordance with institutional and ethical guidelines and subject to a material transfer agreement. The corresponding author typically responds within four weeks.

## Abbreviations

AAV: Adeno-associated virus
BD: Best disease
BE: Base editing
BEST1: Bestrophin-1
BVMD: Best vitelliform macular dystrophy
Cas: CRISPR-associated protein
cDNA: Complementary DNA
CRISPR: Clustered regularly interspaced short palindromic repeats
DSBs: Double-strand breaks
FACS: Fluorescence-activated cell sorting
gDNA: Genomic DNA
HEK293T cells: Human embryonic kidney cells, transformed with large T antigen
hiPSC-RPE: Human induced pluripotent stem cell-derived retinal pigment epithelium
ICC: Immunocytochemistry
NMD: Nonsense-mediated mRNA decay
PAM: Protospacer adjacent motif
PE: Prime editing
PBS: Phosphate-buffered saline
RNP: Ribonucleoprotein complex
RPE: Human retinal pigment epithelium
RT: Reverse transcription
SDS-PAGE: SDS-polyacrylamide gel electrophoresis
sgRNA: Single guide RNA
SpCas9: Streptococcus pyogenes Cas9
WB: Western blotting
WGS: Whole genome sequencing
YFP: Yellow fluorescent protein
