# Supplementary material for: Allele-specific suppression of pathogenic bestrophin-1 transcripts by CRISPR/Cas9-mediated genome editing
Source: Genome Med. 2026 Apr 20;18:45. doi: 10.1186/s13073-026-01649-3 (PMC13104400; doi:10.1186/s13073-026-01649-3)
Supplement: Supplementary file 1 — Additional file 1. Table S1-S4. [file 13073_2026_1649_MOESM1_ESM.pdf]

Table S1: Oligonucleotide sequences for sgRNAs and corresponding target sequences

Primer name	5'-3' Sequence	Application
sgRNA_A243V_II_20nt_F<br>sgRNA_A243V_II_20nt_R	CACCGacaccaccacagtcaccacc<br>AAACggtggtgactgtggtggtgtC	Cloning into the BbsI interface of the px330_mcherry vector
sgRNA_A243V_II_18nt_F<br>sgRNA_A243V_II_18nt_R	CACCGaccaccacagtcaccacc<br>AAACggtggtgactgtggtggtC	
sgRNA_A243V_II_17nt_F<br>sgRNA_A243V_II_17nt_R	CACCGccaccacagtcaccacc<br>AAACggtggtgactgtggtggC	
sgRNA_I295del_20nt_F<br>sgRNA_I295del_20nt_R	CACCGgcagagcagctcaacccctt<br>AAACaaggggttgagctgctctgcC	
sgRNA_I295del_18nt_F<br>sgRNA_I295del_18nt_R	CACCGagagcagctcaacccctt<br>AAACaaggggttgagctgctctC	
sgRNA_I295del_17nt_F<br>sgRNA_I295del_17nt_R	CACCGgagcagctcaacccctt<br>AAACaaggggttgagctgctcC	
sgRNA_#1_R218C-T-20nt_F<br>sgRNA_#1_R218C-T-20nt_R	CACCGaacaccttgtgtactcagtg<br>AAACcactgagtacacaaggtgttC	
sgRNA_#1_R218C-T-19nt_F<br>sgRNA_#1_R218C-T-19nt_R	CACCGacaccttgtgtactcagtg<br>AAACcactgagtacacaaggtgtC	
sgRNA_#1_R218C-T-18nt_F<br>sgRNA_#1_R218C-T-18nt_R	CACCGcaccttgtgtactcagtg<br>AAACcactgagtacacaaggtgC	
sgRNA_#1_R218C-T-17nt_F<br>sgRNA_#1_R218C-T-17nt_R	CACCGaccttgtgtactcagtg<br>AAACcactgagtacacaaggtC	
sgRNA_#2_R218C-T-20nt_F<br>sgRNA_#2_R218C-T-20nt_R	CACCGgtgtccacactgagtacaca<br>AAACtgtgtactcagtgtggacacC	
sgRNA_#2_R218C-T-19nt_F<br>sgRNA_#2_R218C-T-19nt_R	CACCGtgtccacactgagtacaca<br>AAACtgtgtactcagtgtggacaC	
sgRNA_#2_R218C-T-18nt_F<br>sgRNA_#2_R218C-T-18nt_R	CACCGgtccacactgagtacaca<br>AAACtgtgtactcagtgtggacC	
sgRNA_#2_R218C-T-17nt_F<br>sgRNA_#2_R218C-T-17nt_R	CACCGtccacactgagtacaca<br>AAACtgtgtactcagtgtggaC	
sgRNA_#1_R218C-19nt(19)_A_C_F<br>sgRNA_#1_R218C-19nt(19)_A_C_R	CACCGccaccttgtgtactcagtg<br>AAACcactgagtacacaaggtggC	
sgRNA_#1_R218C-19nt(19)_A_G_F<br>sgRNA_#1_R218C-19nt(19)_A_G_R	CACCGgcaccttgtgtactcagtg<br>AAACcactgagtacacaaggtgcC	
sgRNA_#1_R218C-19nt(19)_A_T_F<br>sgRNA_#1_R218C-19nt(19)_A_T_R	CACCGtcaccttgtgtactcagtg<br>AAACcactgagtacacaaggtgaC	
sgRNA_#1_R218C-19nt(18)_C_A_F<br>sgRNA_#1_R218C-19nt(18)_C_A_R	CACCGaaaccttgtgtactcagtg<br>AAACcactgagtacacaaggtttC	
sgRNA_#1_R218C-19nt(18)_C_G_F<br>sgRNA_#1_R218C-19nt(18)_C_G_R	CACCGagaccttgtgtactcagtg<br>AAACcactgagtacacaaggtctC	
sgRNA_#1_R218C-19nt(18)_C_T_F<br>sgRNA_#1_R218C-19nt(18)_C_T_R	CACCGataccttgtgtactcagtg<br>AAACcactgagtacacaaggtatC	
A243V_T241N-EcoRI-F<br>A243V_T241N-BamHI-R	gaattctaagtgtgcaagtcagaacaagg<br>ggatccagtgcagtggcacgatctc	Cloning of the ~500 bp target sequence into the pCAG-EgxxFP vector and analysis of indel frequencies at the on-target locus in hiPSC-RPEs
I295del-EcoRII-F<br>I295del-BamHII-R	gaattcggaaggactggctcagaaga<br>ggatccgcctgtgaaatggggagat	
Q238R-EcoRI-F<br>Q238R-BamHII-R	gaattcacagccaggaatggaccatag<br>ggatccactcctgacctcaggtgatcc	

Note: Uppercase letters indicate the restriction site sequences used for ligation into the px330-mcherry expression vector. Lowercase letters represent the sgRNA-specific target sequence. Nucleotides highlighted in red denote introduced mutations or base substitutions.

Table S2. Overview of the hiPSC lines used in the current study

HiPSC lines generated from patients and healthy controls*					
**IPSC line**	**Amino acid substitution**	**Nucleotide substitution**	**Alias (Clone number)**	**sex**	**Clinical findings**
$MK_{control}$	+/+	-	MK (#27b)	m	healthy
$AM_{control}$	+/+	-	AM (#13)	w	healthy
$MO_{+/R218C}$	+/R218C	652C>T	MO (#214)	m	Best disease
$SK_{+/A243V}$	+/A243V	728C>T	SK (#16)	m	Best disease
$AP_{+/I295del}$	+/I295del	884_886delTCA	AP (#187)	m	Best disease
$MD_{+/I295del}$	+/I295del	884_886delTCA	MD (#18)	m	Best disease

HiPSC lines generated following electroporation of the sgRNA/Cas9 complex			
**IPSC line**	**Parental cell line**	**Insertion/deletion**	**Consequence after Cas9 treatment**
$AP_{crispr\#16}$	$AP_{+/I295del}$	11 bp deletion	DSB-induced frameshift
$AP_{crispr\#19}$	$AP_{+/I295del}$	In/del	DSB-induced frameshift
$MD_{crispr\#2}$	$MD_{+/I295del}$	8 bp deletion	DSB-induced frameshift
$MD_{crispr\#22}$	$MD_{+/I295del}$	8 bp deletion	DSB-induced frameshift
$MD_{+/I295del\_}$#1	$MD_{+/I295del}$	no	no
$MD_{+/I295del\_}$#3	$MD_{+/I295del}$	no	no

*A detailed clinical description of the patients can be found in Milenkovic et al. (2015) and Nachtigal et al. (2020)

Table S3. Overview of hiPSC clones used for each experiment

**Experiments**	**hiPSC clones used**
Quantification of indel frequencies of Sanger-sequenced amplicons from CRISPR/SpCas9-treated hiPSC-RPE cells	$MO_{+/R218C}$, $SK_{+/A243V}$, $AP_{+/I295del}$
Off-Target analysis via deep sequencing	$MO_{+/R218C}$, $SK_{+/A243V}$, $AP_{+/I295del}$
Confocal immunofluorescence imaging	$MK_{control}$, $AM_{control}$, $AP_{+/I295del}$, $MD_{+/I295del}$, $AP_{crispr\#16}$, $AP_{crispr\#19}$, $MD_{crispr\#2}$, $MD_{crispr\#22}$, $MD_{+/I295del}$_#1, $MD_{+/I295del}$_#3
Quantitative analysis of BEST1 protein expression	$MK_{control}$, $AM_{control}$, $AP_{+/I295del}$, $MD_{+/I295del}$, $AP_{crispr\#16}$, $AP_{crispr\#19}$, $MD_{crispr\#2}$, $MD_{crispr\#22}$, $MD_{+/I295del}$_#1, $MD_{+/I295del}$_#3
Quantification of YFP fluorescence intensities in the halide transport assay	$MK_{control}$, $AM_{control}$, $AP_{+/I295del}$, $MD_{+/I295del}$, $AP_{crispr\#16}$, $AP_{crispr\#19}$, $MD_{crispr\#2}$, $MD_{crispr\#22}$, $MD_{+/I295del}$_#1, $MD_{+/I295del}$_#3
Analysis of mRNA expression of pluripotency markers	$MK_{control}$, $AM_{control}$, $AP_{+/I295del}$, $MD_{+/I295del}$, $AP_{crispr\#16}$, $AP_{crispr\#19}$, $MD_{crispr\#2}$, $MD_{crispr\#22}$, $MD_{+/I295del}$_#1, $MD_{+/I295del}$_#3

Table S4. Gene Identifiers, Primer Sequences, and Amplicon Sizes Used for RT-PCR analysis

Gene	Primer Sequence (5` - 3`)	Product Size (BP)	GenBank Accession ID
**SOX2**	Forward: ACACCAATCCCATCCACACT<br>Reverse: GCAAACTTCCTGCAAAGCTC	224	NM_003106.2
**FN1**	Forward: AATATCTCGGTGCCATTTGC<br>Reverse: AAAGGCATGAAGCACTCAAT	193	NM_212482.4
**GAPDH**	Forward: ATCGTGGAAGGACTCATGACC<br>Reverse: AGCGCCAGTAGAGGCAGGGAT	132	NM_002046.7
**RPE65**	Forward: CAATGGGTTTCTGATTGTGGA<br>Reverse: CCAGTTCTCACGTAAATTGGCTA	142	NM_000329.2
**BEST1**	Forward: CAGTACGAGAACCTGCCGTG<br>Reverse: GGTAGGCTCAGTTTCTCCAA	245	NM_004183.3
**RLBP1**	Forward: agctcacaaccaaggaccat<br>Reverse: tctctcgttcagctcatcctt	95	NM_000326.4

Primers were designed with online software Primer3.
