# Supplementary material for: Allele-specific suppression of pathogenic bestrophin-1 transcripts by CRISPR/Cas9-mediated genome editing
Source: Genome Med. 2026 Apr 20;18:45. doi: 10.1186/s13073-026-01649-3 (PMC13104400; doi:10.1186/s13073-026-01649-3)
Supplement: Supplementary file 3 — Additional file 3. Table S5. Genome-wide off-target analysis in CRISPR/Cas9-edited patient-derived hiPSC-RPE cell lines. [file 13073_2026_1649_MOESM3_ESM.pdf]

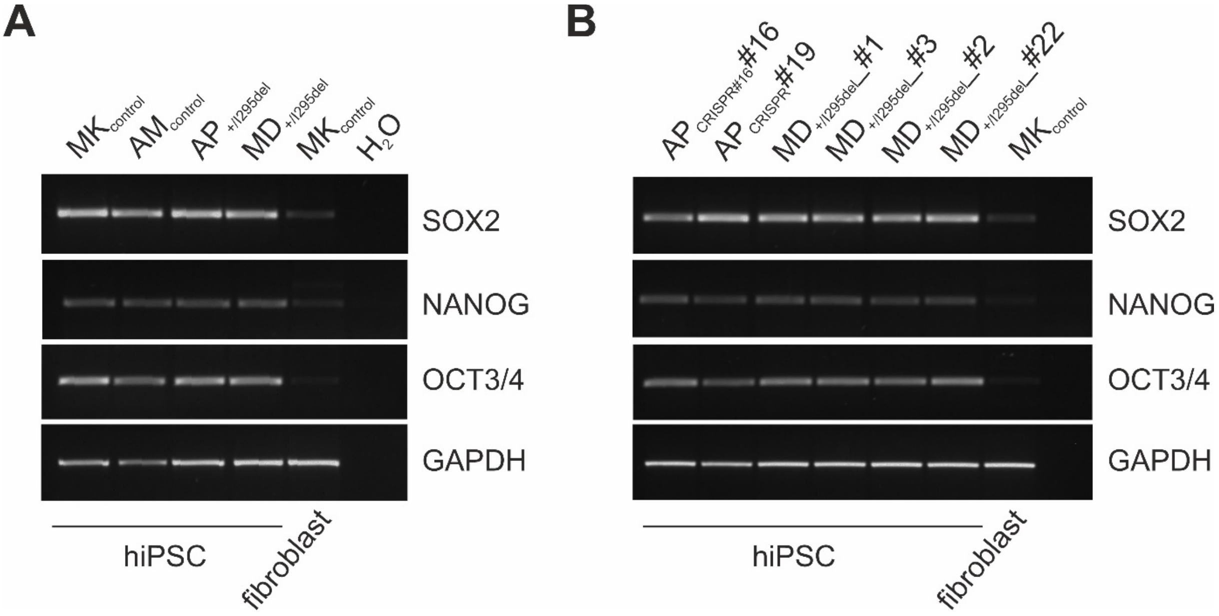

Fig. S1. **Characterization of hiPSCs derived from BD patients, healthy controls and CRISPR/SpCas9-edited cells**

Analysis of mRNA expression of the pluripotency markers *SOX2*, *NANOG*, and *OCT3/4* was performed. (**A**) Expression profiles of the hiPSC lines MK$_{control}$, AM$_{control}$, AP$_{+/I295del}$, and MD$_{+/I295del}$ were compared to fibroblasts of MK$_{control}$, confirming successful reprogramming into hiPSCs. (**B**) After CRISPR/SpCas9 editing of hiPSCs from $_{AP+/I295del}$ and MD$_{+/I295del}$, expression profiles of the edited hiPSC lines (AP$_{crispr\#16}$, AP$_{crispr\#19}$, MD$_{crispr\#1}$, MD$_{crispr\#2}$, MD$_{crispr\#3}$, and MD$_{crispr\#22}$) were compared to fibroblasts, confirming that the edited hiPSCs retained their pluripotent stem cell characteristics. *GAPDH* was used as a housekeeping gene control.

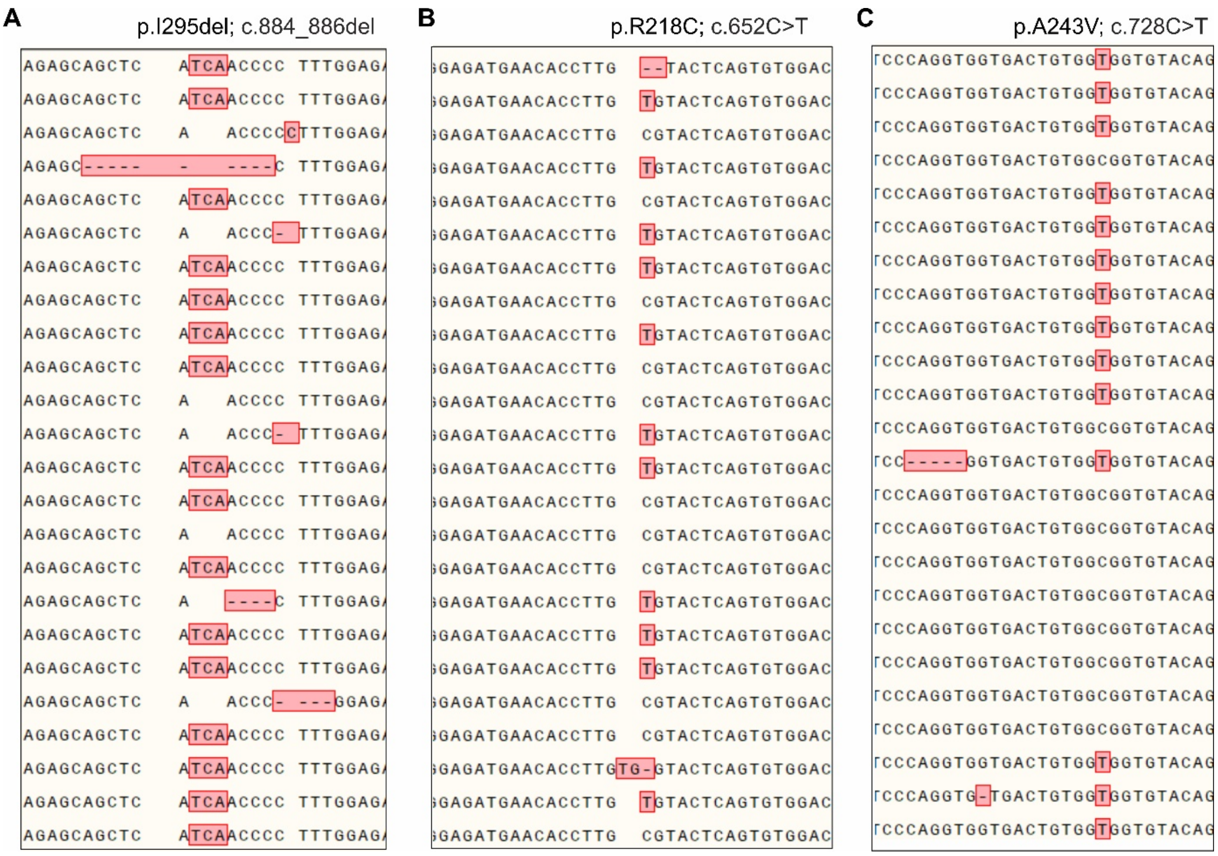

Fig S2. **Representative SnapGene visualization of Sanger-sequenced amplicons from CRISPR/SpCas9-edited hiPSC-RPE cells**

Shown are individual sequencing traces and corresponding SnapGene alignments of PCR amplicons derived from CRISPR/SpCas9-treated hiPSC-RPE cells carrying the indicated heterozygous BEST1 mutations. The edited regions display indel patterns consistent with Cas9-induced double-strand breaks followed by non-homologous end joining (NHEJ) repair. sgRNA and SpCas9 were delivered into patient-derived hiPSC-RPE cells via plasmid transfection. Genomic DNA was extracted from mCherry-positive, FACS-sorted hiPSC-RPE cells and subjected to PCR amplification using primer pairs flanking the target regions. PCR amplicons were subcloned into the pGEM-T Easy vector, and individual clones per transfection were analyzed by Sanger sequencing to quantify indel events. (**A**) 24 clones were analyzed, of which 8 carried the 3 bp deletion TCA (p.(I295del)) including 6 CRISPR-edited clones. (**B**) 24 clones were analyzed, of which 13 carried the mutated T allele (p.(R218C)), including 2 CRISPR-edited clones. (**C**) 24 clones were analyzed, of which 14 carried the mutated T allele (p.(A243V)), including 2 CRISPR-edited clones.

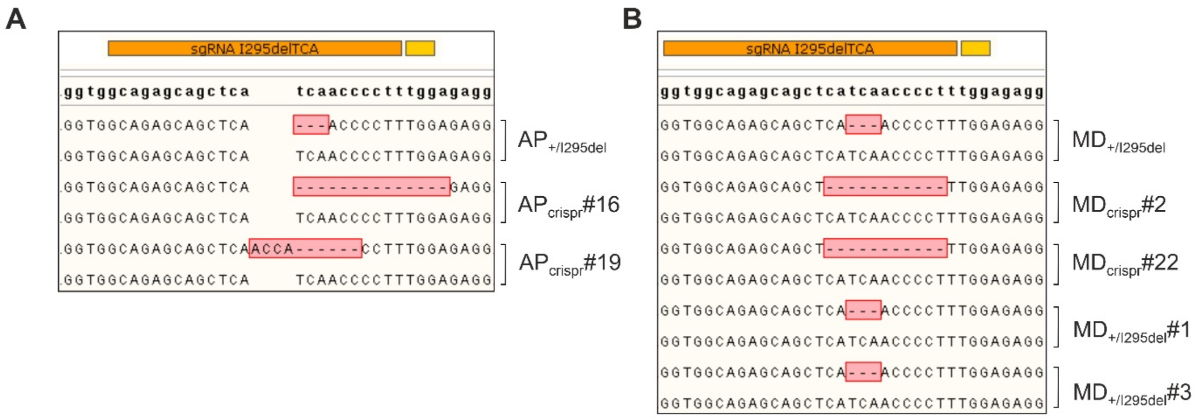

Fig S3. **Indel formation in CRISPR-edited single-cell clones.**

CRISPR/Cas9-mediated genome editing was performed on induced pluripotent stem cells (iPSCs) derived from two patients diagnosed with Best vitelliform macular dystrophy (BVMD), both carrying the heterozygous *BEST1*-I295del mutation. To assess the efficiency and outcome of CRISPR-induced double-strand breaks (DSBs), genomic DNA was extracted from single-cell clones and amplified via PCR using primers flanking the target region of the *BEST1* gene. For allelic discrimination, PCR amplicons were subcloned into a PCR vector and analyzed by Sanger sequencing. Sequence alignments were visualized using SnapGene® software. The CRISPR-modified clones AP_{CRISPR}#16, AP_{CRISPR}#19, MD_{CRISPR}#2, and MD_{CRISPR}#22 exhibited insertions and deletions (indels), specifically introduced at the mutated allele. Detected indels led to frameshift mutations, resulting in the generation of premature stop codons within exon 8 of the BEST1 gene. These findings confirm successful allele-specific editing and support the potential of CRISPR-based approaches to functionally inactivate disease-causing alleles in dominant genetic disorders.

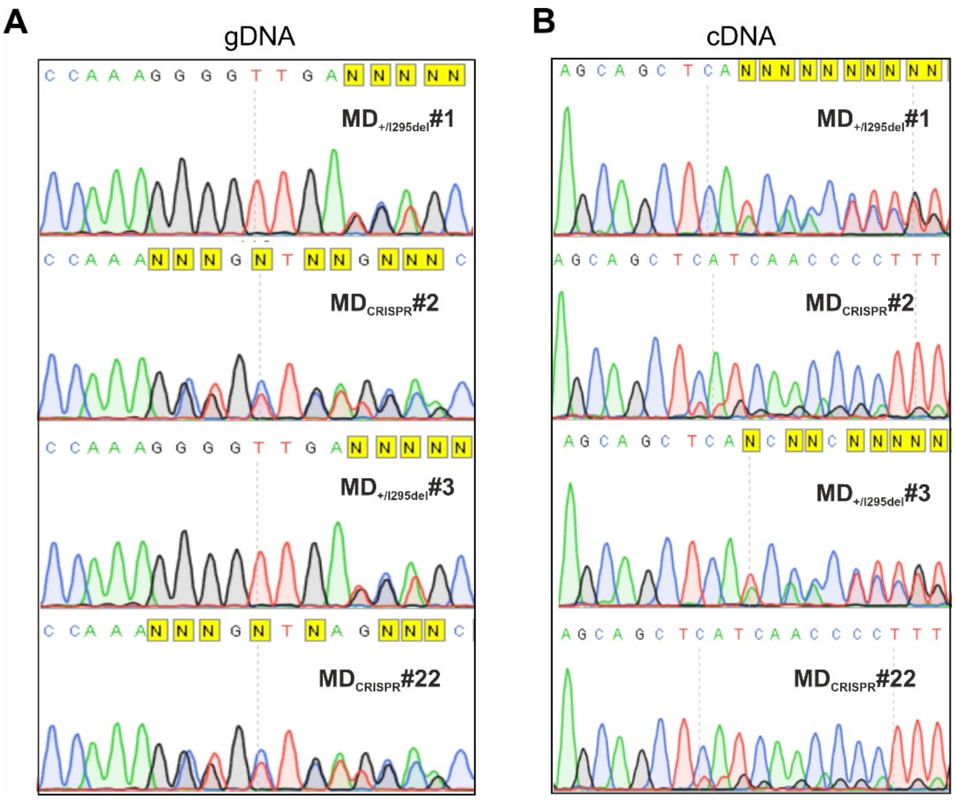

Fig S4: *BEST1* mRNA expression at the BEST1-I295del locus in untreated and CRISPR/SpCas9-treated hiPSC-RPE cell lines.

Sanger sequencing analysis of the *BEST1*-I295del gene locus was performed on both (**A**) genomic DNA (gDNA) and (**B**) complementary (cDNA) obtained from untreated (AP$_{+/I295del}$, MD$_{+/I295del}$#1 and MD$_{+/I295del}$#3) and CRISPR/SpCas9-edited hiPSC-derived RPE cells (AP$_{CRISPR}$#16, AP$_{CRISPR}$#19, MD$_{CRISPR}$#2 and MD$_{CRISPR}$#22), following six weeks of maturation on Transwell filters. Sequence alignments were visualized using SnapGene® software. In the CRISPR-edited cell lines, cDNA sequencing revealed the exclusive presence of the wildtype *BEST1* transcript, while the mutant allele was undetectable at the mRNA level. This observation strongly suggests that the I295del mutant transcript is selectively degraded, likely via the nonsense-mediated mRNA decay (NMD) pathway.
